# Preparation of Molecularly Imprinted Microspheres as Biomimetic Recognition Material for In Situ Adsorption and Selective Chemiluminescence Determination of Bisphenol A

**DOI:** 10.3390/polym10070780

**Published:** 2018-07-16

**Authors:** Yan Xiong, Qing Wang, Ming Duan, Jing Xu, Jie Chen, Shenwen Fang

**Affiliations:** 1 School of Chemistry and Chemical Engineering, Southwest Petroleum University, Chengdu 610500, China; qwang@163.com (Q.W.); jiechen92@163.com (J.C.); swhen@163.com (S.F.); 2 Oil and Gas Field Applied Chemistry Key Laboratory of Sichuan Province, Southwest Petroleum University, Chengdu 610500, China; 3 Liaoning Entry-Exit Inspection and Quarantine Bureau, Dalian 116001, China; jingxu99@163.com

**Keywords:** molecularly imprinted microsphere (MIMS), bisphenol A, in situ adsorption, chemiluminescence determination, selectivity

## Abstract

Bisphenol A (BPA) is an endocrine disrupter in environments which can induce abnormal differentiation of reproductive organs by interfering with the action of endogenous gonadal steroid hormones. In this work, the bisphenol A (BPA) molecularly-imprinted microspheres (MIMS) were prepared and used as biomimetic recognition material for in situ adsorption and selective chemiluminescence (CL) determination of BPA. Through non-covalent interaction, the BPA-MIMS was successfully prepared by Pickering emulsion polymerization using a BPA template, 4-vinylpyridine (4-VP) monomer, ethylene glycol dimethacrylate (EGDMA) cross-linker, and a SiO_2_ dispersion agent. The characterization of scanning electron microscopy (SEM) and energy-disperse spectroscopy (EDS) showed that the obtained MIMS possessed a regular spherical shape and narrow diameter distribution (25–30 μm). The binding experiment indicated BPA could be adsorbed in situ on the MIMS-packing cell with an apparent maximum amount *Q*_max_ of 677.3 μg g^−1^. Then BPA could be selectively detected by its sensitive inhibition effect on the CL reaction between luminol and periodate (KIO_4_), and the inhibition mechanism was discussed to reveal the CL reaction process. The CL intensity was linear to BPA concentrations in two ranges, respectively from 0.5 to 1.5 μg mL^−1^ with a detection limit of 8.0 ng mL^−1^ (3σ), and from 1.5 to 15 μg mL^−1^ with a limit of detection (LOD) of 80 ng mL^−1^ (3σ). The BPA-MIPMS showed excellent selectivity for BPA adsorption and the proposed CL method has been successfully applied to BPA determination in environmental water samples.

## 1. Introduction

Bisphenol A (BPA) is the most widely used bisphenol (BP), commonly used as the chemical building block in the manufacture of polycarbonate plastics [1] and generally as the weakly acidic color developer in the production of thermal papers [2]. However, the leakage of BPA from receipt papers [3] and drinking bottles [4,5] into food and water will result in potential risks on public health. Many studies have shown that BPA is an endocrine disrupter in the environment, which can induce abnormal differentiation of reproductive organs by interfering with the action of endogenous gonadal steroid hormones [6]. As a result, several states in the United States have prohibited BPA from manufacture, sale, or distribution in some consumer products since 2009 [7]. The European Commission [8] and China government restricted the use of BPA in plastic infant feeding bottles in January 2011 and June 2012. The U.S. Food and Drug Administration (FDA) also banned the use of BPA in baby bottles and children’s drinking cups since 2012 [9]. Hence, there is a great need to develop reliable analytical methods for sensitive detection of trace BPA in the fields of environmental monitoring, food safety, and toxicity assessment.

Up to now, a large number of analytical methods have been reported for the determination of BPA, including electrochemistry [10,11,12], chromatography [13,14,15,16], mass spectroscopy [17], chemiluminescence (CL) [18,19], and fluorescence [20,21]. However, these methods for BPA measurement still have some disadvantages, as reported in the literature [22]. For the mentioned methods, chemiluminescence combined with flow-injection analysis shows unique merits of high sensitivity, short analysis time, and simple instrumentation manipulation. However, the poor selectivity of the CL technique means it cannot be used for direct analysis of complicated samples [23]. 

Molecular imprinting technology (MIT) is an attractive technique which has been widely utilized to fabricate artificial tailor-made receptors [23]. With excellent memory of size, shape, and functional groups to template molecules, the resultant molecularly-imprinted polymers (MIPs) show structure predictability, recognition specificity and application universality [24]. The above-mentioned features result in MIPs being employed as biomimetic molecular recognition receptors for a given molecular structure [25].

Among the polymerization methods, traditional bulk polymerization is widely employed for MIPs preparation due to the advantages of rapidity and simplicity [26]. However, the post-treatment of crushing, grinding, and sieving is generally needed for the obtained monolithic polymer by bulk polymerization. This post-treatment procedure is not only time-consuming, but also causes irregular particles and low yields of polymer. To overcome these disadvantages, suspension polymerization [27,28] and precipitation polymerization [29,30] methods were developed to prepare MIPs. However, the MIP microspheres obtained by suspension polymerization have polydisperse particle sizes with large variability, which is not beneficial for the application [31]. Meanwhile, precipitation polymerization uses a large amount of organic solvent and needs very strict reaction conditions with respect to polymerization temperature and stirring speed [32].

A new molecular imprinting method based on Pickering emulsion polymerization (PEP) was developed by Shen et al. [33]. The phenomenon of Pickering emulsion was firstly described by Pickering in 1907 [34], in which solid particles could be employed to stabilize emulsion droplets either in oil-in-water (O/W) or water-in-oil (W/O). By locating the stabilizing particles at the interface between the two immiscible liquids, the coalescence was successfully prevented and the droplets were stabilized [35,36]. By combining Pickering emulsion polymerization (PEP) with MIT, the resulted molecularly imprinted microspheres (MIMS) could achieve high yields of polymer and good control of particle sizes. Meanwhile, with an optimal combination of hydrophobic and electrostatic interactions, this PEP-MIT method can lead to successful formation of molecularly imprinted sites in a continuous water phase [37]. The PEP-MIT based MIMS have been successfully applied as λ-cyhalothrin recognition element [38], peptides catalyst [39], drugs β-receptor blocker [40], solid phase extraction material [41], etc. 

This work was aimed to develop an improved CL method for in situ and selective determination of BPA using MIMS as biomimetic recognition material. The BPA-MIMS was fabricated by Pickering emulsion polymerization through non-covalent interaction with the BPA template, 4-vinylpyridine (4-VP) monomer, ethylene glycol dimethacrylate (EGDMA) cross-linker, and the SiO_2_ dispersion agent. Particles of BPA-MIMS were packed into the CL detection cell and used to adsorb BPA on-line. Then the adsorbed BPA can be detected in situ by producing a sensitive inhibition effect on the CL reaction between luminol and periodate (KIO_4_). After the CL reaction, the absorbed BPA was destroyed and taken away by the flow solution with the cavities left on the MIMS for the adsorption and detection of the next sample. The BPA-MIMS showed excellent selectivity for BPA adsorption and has been successfully applied to CL determination of BPA in water samples.

## 2. Materials and Methods

### 2.1. Reagents and Materials

4-Vinylpyridine (4-VP) and luminol were obtained from Aladdin Biochemical Technology Inc., Ltd. (Shanghai, China), ethylene glycol dimethacrylate (EGDMA) was purchased from Letai Chemical Reagent Company (Tianjin, China), and the other reagents were obtained from Kelong Chemical Reagent Company (Chengdu, China). All the reagents were of analytical grade and used as received except EGDMA. EGDMA was distilled to eliminate radical inhibitors. Wahaha^®^ purified water (Wahaha, Hangzhou, China) was used for the preparation of solutions and throughout the experiments.

A total of 100 μg mL^−1^ BPA stock solution was prepared by dissolving 0.05 g BPA in 500 mL of water. A total of 1.0 × 10^−3^ mol L^−1^ luminol stock solution was prepared by dissolving 0.0886 g of luminol in 10 mL of NaOH (1.0 mol L^−1^) and diluted to 500 mL. A total of 1.0 × 10^−3^ mol L^−1^ potassium periodate (KIO_4_) stock solution was prepared by dissolving 0.1150 g of KIO_4_ in 500 mL of water. All the solutions were stored in a refrigerator and protected from lighting. 

### 2.2. Preparation of BPA-MIMS Specifically Targeted BPA

The BPA-MIMS molecularly imprinted polymers applied for solid phase extraction (SPE) procedure were prepared based on Pickering emulsion polymerization according to [41]. Traditionally, the preparation of BPA-MIMS mainly includes the following steps: (i) preparation of the Pickering emulsion; (ii) free-radical polymerization; and (iii) solvent extraction. 

Specifically, the water phase used for the emulsion preparation mainly was shaped by solid stabilizing SiO_2_ nanoparticles (30 mg), mixed with a triton X-100 water solution (0.2%, 10 mL) in a 20 mL thick walled glass tube and sonicated for 10 min. On the other hand, the oil phase was composed of the template BPA (0.228 g, 1 mmol), the functional monomer 4-VP (0.42 mL, 4 mmol), the cross-linking monomer EGDMA (20 mmol, 3.8 mL), and initiator AIBN (40 mg), and then these reagents were dissolved in 3.6 mL of the pore-forming agent toluene in a 10 mL thick-walled glass tube to prepare the pre-polymerization solution, and following sonication for 1 min was carried out to homogenize the mixture in the oil phase. After the addition of 4 mL of pre-polymerization solution to the water phase, the mixture was shaken by intense agitation, a stable Pickering emulsion was obtained when no coalescence of the oil droplets could be observed in 2 h.

The free radical polymerization of the monomer 4-VP in the Pickering emulsion was conducted in a water bath at 70 °C for 16 h, polymer-silica microsphere composite was obtained. The mixture was cooled to room temperature, and the supernatant in the mixture was removed. The sinking microspheres, evenly coated with the polymer-silica microsphere powders, were isolated by filtration, were washed with water, ethanol, and acetone in sequence, and dried overnight under vacuum. Then the polymer-silica composite was cleaned by dipping into a hydrofluoric acid (40%) at room temperature for 12 h to remove the silica particles from the surface.

**Scheme 1:**
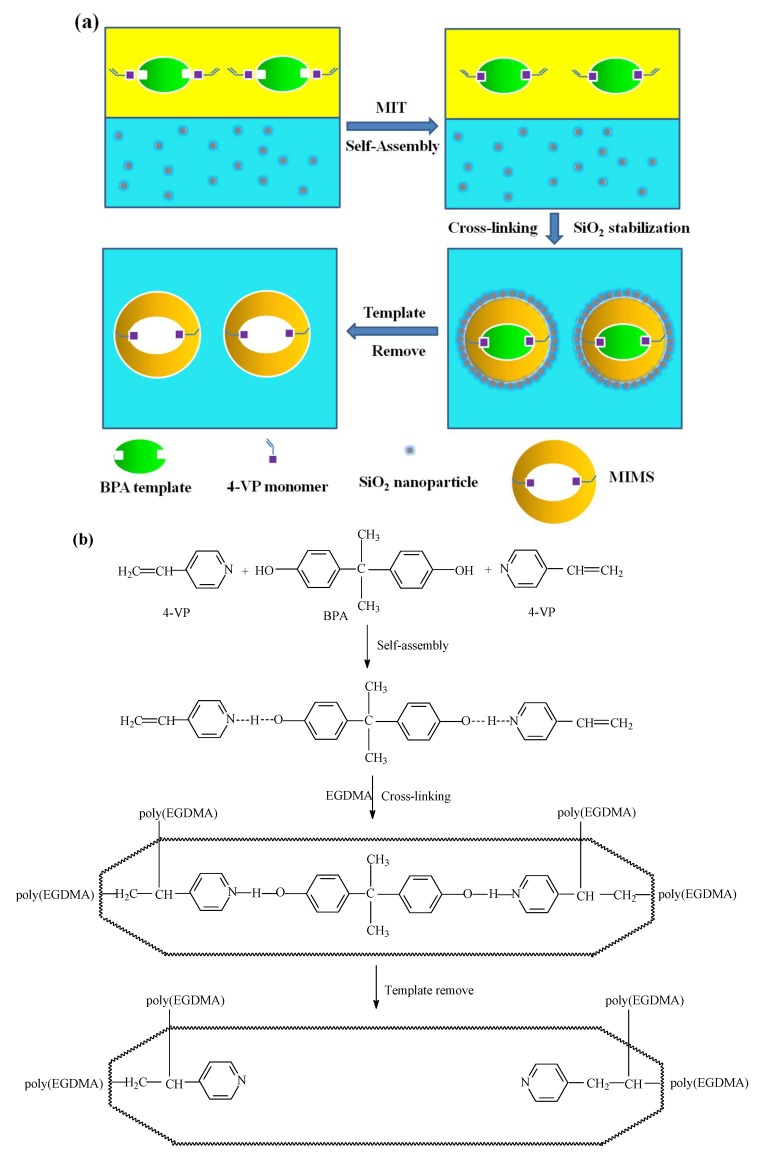
(**a**) Schematic illustration of the synthesis of molecularly imprinted microspheres with self-assembly at Oil/Water interfaces in a SiO_2_ NP-stabilized Pickering emulsion; and (**b**) the overall scheme of the BPA-MIMS fabrication process.

The final solvent extraction of the BPA template molecules was achieved using a Soxhlet extractor in a methanol solution containing 10% (vol %) acetic acid as the extraction solvent for 24 h. The overall scheme of BPA-MIMS fabrication process was summarized in Scheme 1a,b. For the preparation of non-imprinted microsphere (NIMS), the similar procedure was adopted except that the pre-polymerization solution without BPA was used and the Soxhlet extraction step was omitted. 

### 2.3. Characterization

The fluorescence spectra for binding studies were measured by a fluorescence spectrometer (LS55, Perkin Elmer, Connecticut, CT, USA). Solutions were placed in a quartz cuvette with 1 cm path length. The fluorescence emission spectra were measured by setting excitation wavelength at 279 nm. The scan speed was chosen to be 500 nm min^−1^ and the widths of the excitation and the emission slits were both set to 10 nm.

Scanning electron microscopy (SEM) images were recorded using a field-emission scanning electron microscope (FESEM, EV0 MA15, Carl Zeiss, OB Cohen, Germany) at an acceleration voltage of 20 kV. All samples were sputter-coated with gold using an E1045 Pt-coater (Carl Zeiss, Germany) before SEM observation. Elemental analysis was conducted with an energy dispersive X-ray spectrometer (EDS) equipped in the EV0 MA15 FESEM at an accelerating voltage of 20 kV.

The optical microscope images of the Pickering emulsion were taken using a laser scanning confocal microscope (LSCM, Eclipse Ti, Nikon, Tokyo, Japan) with an excitation wavelength of 488 nm. The zeta potential and dynamic light scattering (DLS) measurements were performed on a NANO ZS apparatus equipped with the DTS Ver. 4.10 software package (Malvern Instruments Ltd., Worcestershire, Malvern, UK). Fourier transform infrared spectroscopy (FTIR, Nicolet 6700, Thermo Scientific, Massachusetts, MA, USA) was used to analyze the chemical structures and compositions of the MIMS.

### 2.4. Chemiluminescence Measurements

Chemiluminescence measurements were carried out using the flow system shown in Figure S1. Solutions were delivered by two peristaltic pumps (HL-2B, Shanghai Huxi, Shanghai, China). PTFE tubing (0.8 mm i.d.) was used to connect the components. A six-way injection valve (XP-206, Shanghai Kincaid Analytical Instrument Co., Ltd., Shanghai, China) was used for solution sampling. An ultra-weak chemiluminescence measuring instrument (RFL-1, Xi’an Remex Analysis Instrument Co. Ltd., Xi’an, China) equipped with an lp21 photomultiplier tube (PMT, Hamamatsu, Japan) was used to detect the CL signal and the data was processed with computerized data processing software. Fifty milligram particles of BPA-MIMS were packed into a glass tube (2 mm inner diameter (i.d.) × 2 cm length) used as the CL detection cell and positioned in front of the PMT detector. The procedure for chemiluminescence determination of BPA was summarized as follows:

Step 1 (For BPA on-line adsorption): pump 1 is stopped and BPA solution was delivered to flow through the BPA-MPMS cell by pump 2. As a result, BPA was selectively adsorbed in situ on the MIMS.

Step 2 (For BPA in situ CL determination): after a proper adsorption time, the BPA was washed with NaOH solution by using the six-way injection valve. At the same time, the emerging stream of KIO_4_-luminol CL reagents was delivered by pump1 and flowed through the BPA-MPMS cell. As a result, BPA could react with the CL reagents under NaOH conditions, producing an inhibition effect on CL emissions and be sensitively detected. 

Step 3 (For BPA-MIMS cleaning): when the CL intensity of the reaction came to be blank, pump 1 was stopped immediately and the water carrier delivered by pump 2 flowed through the cell to clean the MPMS cavities for the next BPA adsorption and determination. 

### 2.5. Binding Measurements

The binding properties of MPMS to BPA were studied by the batch method and the dynamic method. The amount of BPA left in the solution after rebinding was detected through fluorescence measurement. 

In a typical rebinding experiment for batch method, six portions of 20.0 mL of different concentration BPA solutions (10, 20, 30, 40, 50, and 60 μg mL^−1^) were added to six pieces of 10 mg washed and dried MIMS and placed in the tubes, respectively. Then the suspensions were sealed and were oscillated for 2 h at room temperature to ensure equilibration. After centrifuging at 3000 rpm for 15 min, the supernatants were respectively taken out and put into the quartz cell to detect the concentration of free BPA by measuring the fluorescence. The amount of BPA bound to the MIMS polymer was calculated by subtracting the concentration of free BPA from the initial BPA concentration. The data obtained was used to draw the adsorption isotherm for Scatchard analysis according to the Equations (1) and (2) [42,43]:

(1) $$Q\;=\;\frac{\left(C_{0}-C_{R}\right)v}{m}\;$$

(2) $$\;\frac{Q}{C_{R}}\;=\;-\frac{Q}{K_{d}}+\frac{Q_{\max}}{K_{d}}$$

where *C*_0_ and *C*_R_ are the initial and final BPA concentrations, *v* is the sample volume, *m* is the mass of the used BPA-MIMS, *Q* is the amount of BPA adsorbed to MIMS at equilibrium, *Q*_max_ is the apparent maximum amount of BPA adsorbed by MIMS, and *K*_d_ is the equilibrium dissociation constant.

For the dynamic method, seven portions of 20.0 mL BPA solution with the same concentration (10 μg mL^−1^) were added to seven pieces of 10 mg washed and dried MIMS, respectively. The suspensions were sealed and were oscillated at room temperature to ensure equilibration. Then one sample was taken out at a regular interval to detect the concentration of free BPA in the supernatant by measuring fluorescence. The data obtained was used to draw the kinetic adsorption curve for dynamic analysis.

### 2.6. Analytical Evaluation of the Proposed Method

In order to evaluate the proposed method, serials of analytical performances, including analytical linear range and limit of detection (LOD), repeatability and response time, interference study, and recovery testing were investigated for BPA analysis in detail. Meanwhile, the possible CL reaction mechanism was discussed and this method was applied to BPA determination in several different real water samples. 

## 3. Results

### 3.1. BPA-MIMS Binding Assays

**Figure 1:**
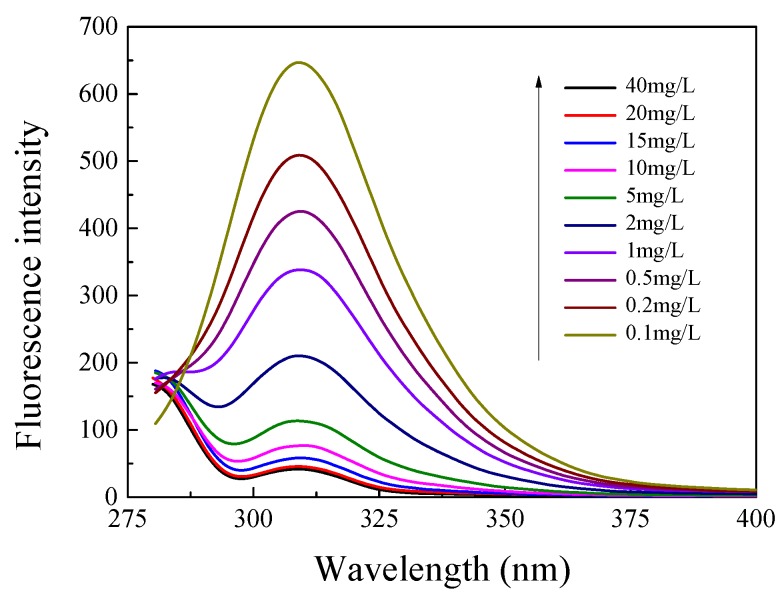
The typical fluorescence responses to different BPA concentrations.

For binding assays, the concentration of free BPA in the supernatant was detected by measuring BPA fluorescence emission at λ_em_ = 309 nm with λ_ex_ = 279 nm. The typical fluorescence responses to different BPA concentrations are shown in Figure 1. 

**Figure 2:**
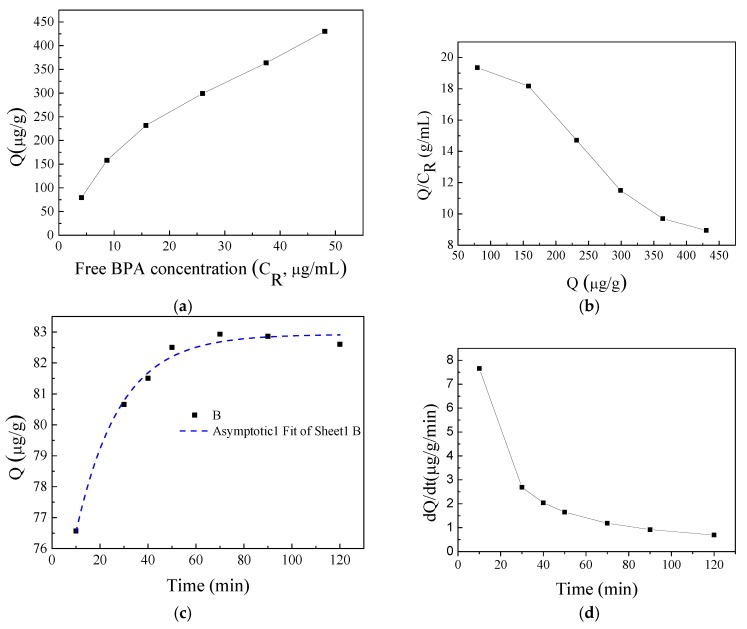
(**a**) The Langmuir adsorption isotherm curve of MIMS with equilibration time of 2 h; (**b**) Scatchard plot to estimate the binding nature of MIMS with equilibration time of 2 h; (**c**) kinetic adsorption curve for dynamic analysis of 10 μg mL^−1^; and (**d**) adsorption rate of MIMS with time changing.

The data obtained from batch-type method were plotted according to the Scatchard equation and shown in Figure 2a,b. The equilibration time of the suspensions for the batch-type experiment was 2 h, as illustrated above in Section 2.5. This result was also confirmed by the dynamic experiment, as shown in Figure 2c, which indicated the adsorption and equilibrium time was about 50 min. From the Scatchard analysis of Figure 2b, the data plotting showed two linear fittings during the analysis BPA concentration range. The first linear regression equation for the straight line is $\frac{Q_{1}}{C_{R}}\;=\;-0.0367Q_{1}+22.976$ and the second linear regression equation for the straight line is $\frac{Q_{2}}{C_{R}}\;=\;-0.0195Q_{2}+17.138.$ This result indicated there are two types of binding sites on MIMS for BPA. Then, according to Equation (2), the equilibrium dissociation constant *K*_d_ and the apparent maximum amount *Q*_max_ were estimated to be *K*_d1_ = 119.36 μmol L^−1^ and *Q*_max1_ = 12.01 μmol/g for the lower affinity binding sites and *K*_d2_ = 224.63 μmol L^−1^ and *Q*_max2_ = 16.83 μmol/g for the higher affinity binding sites.

The different binding characteristics of MIMS were mainly caused by the molecular structure of BPA. As shown in Scheme 1, BPA has two phenolic hydroxyl groups which can react with MAA. Hence, there are two binding sites produced on the MIMS after the extraction of BPA. When the BPA concentration is low, the subsequent equilibrium constant and apparent maximum amount of BPA adsorption are smaller because fewer BPA molecules exist in the low BPA concentration. However, when BPA concentration is high, the subsequent equilibrium constant and apparent maximum amount of BPA adsorption are higher because a much greater amount of BPA molecules exist in the solution.

For the dynamic method, the concentration of free BPA in the supernatant was detected by measuring the fluorescence to evaluate the adsorption rate and adsorption time. The data obtained was used to draw the kinetic adsorption curve for dynamic analysis. As shown in Figure 2c, the adsorption and equilibrium time for 10 μg mL^−1^ was about 50 min. The dynamic kinetic process can be evaluated by an asymptotic fitting model according to the following equation with *R*^2^ = 0.98:

*y* = 82.923 − 11.032 × 0.947^t^ (3)

where y is the absorption amount and t is the absorption time. As a result, the kinetic rate constant *k* can be obtained to be 0.947 min^−1^ from Equation (3). Meanwhile, as shown in Figure 2d, the adsorption rate was decreased with the adsorption time. During the first 30 min, there is a rapid adsorption of BPA on the MIMS. Then the adsorption slowed down after 30 min.

### 3.2. Synthesis and Characterization of BPA-MIMS

**Figure 3:**
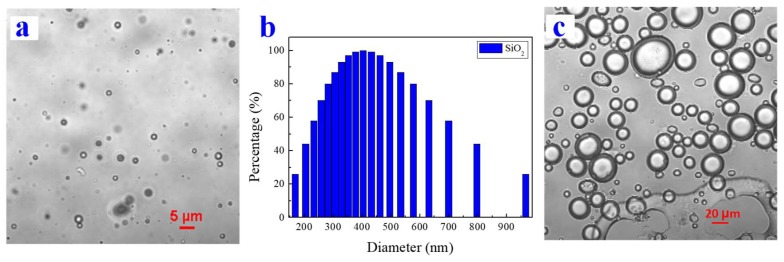
(**a**) Optical image of SiO_2_ NPs; (**b**) DLS measurement for size distribution; and (**c**) an optical image of emulsion droplets after locating the silica NPs at the interface.

In this work, the BPA-MIMS was prepared with non-covalent interactions through the typical Pickering emulsion polymerization and the process was shown in Scheme 1a. BPA was used as a template, 4-VP was used as monomer, and EGDMA was used as a cross-linker for BPA-MIMS polymerization with non-covalent interactions within the organic core. SiO_2_ nanoparticles (NPs) with proper hydrophilic-hydrophobic properties were adopted to stabilize the emulsion at oil/water interfaces. The dynamic light scattering (DLS) indicated the SiO_2_ NPs had an average zeta potential of approximately −20.36 mV with excellent stability and an average diameter of approximately 400 nm with the polydispersity index (PDI) of 0.296, indicative of a good dispersibility. The optical microscope image of the SiO_2_ NPs was shown in Figure 3a and the diameter distribution was shown as Figure 3b. The PDI result of the DLS in this work is consistent with the literature [44], which reported the interfacial polymerization of dopamine in a Pickering emulsion by the use of SiO_2_ NPs.

By locating the stabilizing silica NPs at the interface, the coalescence was successfully prevented and the droplets were stabilized. As shown in Figure 3c, most of the emulsion droplets were spherical in shape with a diameter range of 20−30 μm. In addition, there was no observation of the DLS signal for the water phase above the emulsion, which indicated that there was no free SiO_2_ NPs in the water phase. This was because nearly all of the silica particles took part in the emulsion process and were adsorbed by the droplet interfaces. 

**Figure 4:**
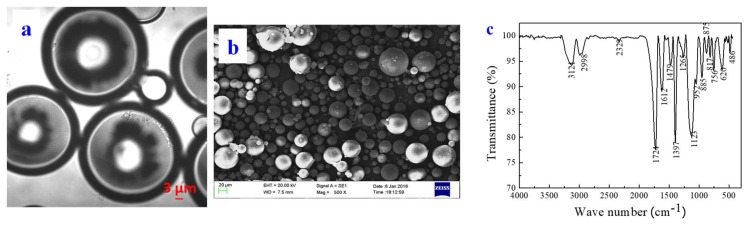
(**a**) The surface morphologies of the dried MIMS examined using FESEM; (**b**) the surface morphologies of the MIMS examined using LSCM; and (**c**) FTIR spectra of the synthesized MIMS.

The surface morphologies of the dried BPA-MIMS were examined using FESEM and LSCM, which are shown in Figure 4a,b, respectively. It could be observed that both FESEM and LSCM indicated that all the MIMS were spherical with an average diameter of approximately 25 μm before SiO_2_ nanoparticle removal. These MIMS showed a good dispersibility without obvious aggregations. Meanwhile, the detached SiO_2_ NPs, which should be derived from the MIMS, were also observed by SEM.

The structural characteristics of the presented MIMS before SiO_2_ removal have been investigated by Fourier transform infrared spectroscopy (FTIR) and spectra were analyzed in detail. Figure 4c presented the FTIR spectra of the MIMS after removing the silica nanoparticles and BPA template. Typically, strong peaks at (1) around 3121 and 756 cm^–1^ corresponding to stretching vibration and out-of-plane bending vibration of C–H bond for C=C–H; (2) 2998 and 2329 cm^–1^ corresponding to asymmetric and symmetric stretching vibration of C–H bond for alkyl; (3) 1724 cm^–1^ corresponding to stretching vibration of C=O for carboxyl; (4) 1612 and 1397 cm^–1^ corresponding to asymmetric and symmetric stretching vibration of C=C for pyridine; (5) 1265 cm^−1^ corresponding to stretching vibration of C–O for carboxyl; (6) 1479 cm^−1^ corresponding to bending vibration of C–H for carboxyl; and (7) 875, 885, and 952 cm^−1^ corresponding to in-plane bending vibration of pyridine ring. These results indicated the EGDMA have been successfully reacted with 4-VP. Meanwhile, there are strong peaks at (1) 1123 cm^–1^ corresponding to asymmetric stretching vibration of Si–O–Si bond; (2) 817 cm^–1^ and around 486 cm^–1^ corresponding to symmetric stretching vibration of Si–O–Si bond, which indicate the presence of SiO_2_ nanoparticles in the MIMS. 

**Figure 5:**
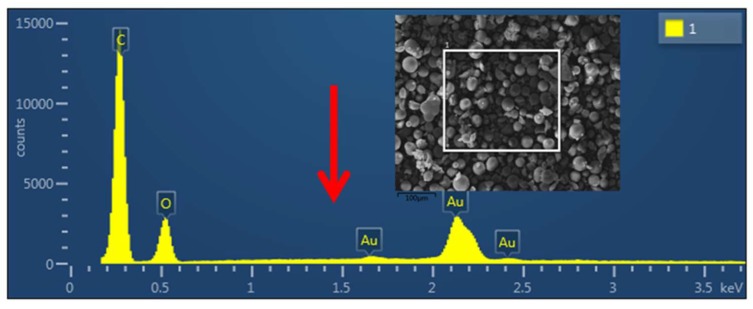
The EDS results of the MIMS after SiO_2_ removing.

The Si apparent concentration was 0.49 before SiO_2_ NPs removal, indicating the presence of Si atoms in the polymer layer. The results showed the presence of Si atoms in the polymer layer. This result was in accord with the result in Figure 4a, in which the detached SiO_2_ NPs were observed. In order to remove the silica NPs, the dried polymer-silica microspheres were transferred into a plastic tube and stirred in HF (40%) at room temperature for 12 h. The obtained MIMS was filtrated, washed with water to be neutral and then rinsed with ethanol. The EDS and SEM results result for the MIMS after SiO_2_ NPs removal was shown in Figure 5. The EDS result indicated the Si apparent concentration changed to be 0 after SiO_2_ NPs removing, suggesting that almost all of the silica NPs have been removed from the BPA-MIMS interfaces after the treatment. Meanwhile, the SEM in Figure 5 showed the shape of the MIMS did not change obviously, indicating that the silica NPs were effective for the BPA-MIMS formation.

### 3.3. CL Instrument Condition Choice and CL Measurement Condition Optimization 

#### 3.3.1. Condition Choice of the CL Instrument

First, the negative high voltage of the PMT equipped on the CL instrument was investigated. Generally speaking, the detection sensitivity would increase with the increasing of the PMT’s negative high voltage. However, the noise would also increase at the same time. As a result, a negative high voltage of 600 V was chosen for the PMT for the following determination with a high signal-to-noise ratio. 

Then the flow speed of the peristaltic pump was also studied. The results showed that the pump speed would have effects on the signal intensity and peak shape. Finally, taking into account the reagent usage and manipulating time together with signal intensity and peak shape, 1.2 and 0.9 mL min^−1^ were chosen as the pump speed for peristaltic pump 1 (P1) and peristaltic pump 2 (P2), respectively. 

#### 3.3.2. Conditions Optimization for CL Measurement

In this work, the CL measurement was based on the CL reaction between KIO_4_ and luminol under a NaOH medium. To obtain a low background emission, luminol and KIO_4_ solutions were mixed together in a proper distance before they flowed through the detection cell. A prohibition CL emission was recorded when BPA reacted with the mixed solution in the flow detection cell and the CL response was proportional to the BPA concentration. To obtain the most sensitive analysis performance for BPA, the conditions for the luminol-KIO_4_-NaOH CL reaction were optimized by measuring the ratio of the CL signal-to-noise (*S/N*) at different reagent concentrations. 

First, KIO_4_ was used as the oxidant for the CL system and its concentration would greatly influence the CL intensity. By fixing the luminol concentration at 2 × 10^−5^ mol L^−1^ and NaOH concentration at 0.4 mol L^−1^, the effect of KIO_4_ concentration was examined from 1 × 10^−5^ to 4 × 10^−4^ mol L^−1^. As shown in Figure S2a, the CL intensity reached a maximum with a better *S*/*N* ratio when KIO_4_ was 5 × 10^−5^ mol L^−1^.

Second, luminol acted as the luminescent reagent for the CL system and its concentration was examined from 5 × 10^−7^ to 4 × 10^−5^ mol L^−1^. As shown in Figure S2b, the CL intensity increased greatly with increasing the concentration of luminol up to 4 × 10^−5^ mol L^−1^. Then the increase of CL intensity is not obvious with further luminol concentration increasing. In order to obtain a preferable CL intensity with a proper luminol concentration, luminol concentration was chosen to be 2 × 10^−5^ mol L^−1^.

Third, the experiment indicated that luminol would produce CL emission just under strong basic conditions, so the NaOH concentration was a key factor for the CL reaction. The effect of NaOH concentration as a medium of luminol was examined over a 0.025–0.8 mol L^−1^ range. As shown in Figure S2c, the CL intensity reached a maximum value when 0.4 mol L^−1^ NaOH was used. A higher concentration of NaOH would result in lower CL intensity. 

In conclusion, the optimal conditions for the CL system were 5 × 10^−5^ mol L^−1^ KIO_4_, 2 × 10^−5^ mol L^−1^ luminol and 0.4 mol L^−1^ NaOH.

#### 3.3.3. Optimum of BPA Adsorption Time

A proper adsorption time was needed for the adsorption of BPA on the MIMS because too short a time may be insufficient for BPA adsorption but too long time would result in time waste and MIMS saturation. By fixing the CL condition of 5 × 10^−5^ mol L^−1^ KIO_4_, 2 × 10^−5^ mol L^−1^ luminol and 0.4 mol L^−1^ NaOH, the adsorption time was investigated from 0 to 7 min. The results in Figure S2d indicated that BPA adsorption amount would increase with the increase of adsorption time and produced a higher prohibition effect on the CL reaction. As a result, as shown, it was observed that the CL intensity decreased with the adsorption time. However, the MIMS adsorption for BPA would come to saturation after a period of time. As shown in Figure S2d, the CL intensity reached constant within 4 min for 2 μg mL^−1^ BPA. As a result, 2 min was chosen as the optimal time to obtain the proper BPA adsorption and avoid the adsorption saturation.

### 3.4. The Analytical Performance for BPA Measurements

#### 3.4.1. Discussion of the Possible CL Reaction Mechanism 

**Figure 6:**
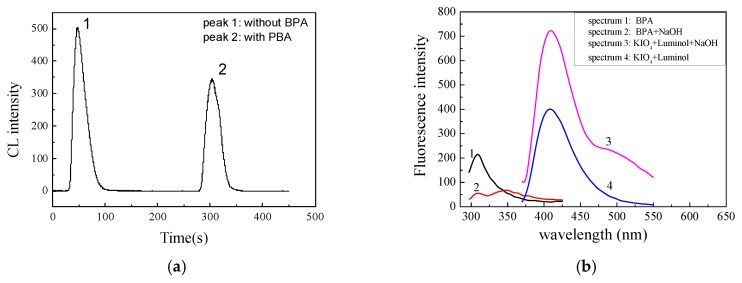
Spectra comparison in the presence and absence of BPA. (**a**) CL spectra; and (**b**) fluorescence spectra.

At the selected conditions, a comparison of the CL spectra and fluorescence spectra in the presence and absence of BPA was shown in Figure 6a,b, respectively. As indicating in Figure 6a, the CL intensity of KIO_4_-luminol system decreased with the addition of BPA. This result confirmed that BPA showed a strong CL inhibition effect on the luminol CL emission. 

The fluorescence result in Figure 6b revealed that a maximum emission wavelength was found at 419 nm, which suggested that the possible emission species was the oxidated 3-aminophthalate (AP^2−*^) and the emitter was 3-aminophthalate (AP^2−^) [45,46]. Since the light emission spectrum was independent of the inhibitor, this result also indicated the prohibition process by BPA was dynamic quenching [47]. Meanwhile, the results showed that the intensity response of BPA had an obvious change with CL reaction, which suggests that BPA has taken part in the CL reaction and interacted with the intermediate radicals produced by the CL reaction. Consequently, the possible light-emitting pathways can be changed and prohibited by BPA as follows:

(4) $${\mathrm{IO}}_{4}{}^{-}+{\mathrm{OH}}^{-}\to {\mathrm{IO}}_{3}{}^{-}+O_{2}{}^{-}+H_{2}O,$$

(5) $${\mathrm{LH}}_{2}+{\mathrm{OH}}^{-}\to {\mathrm{LH}}^{-},$$

(6) $$\;{\mathrm{LH}}^{-}+O_{2}{}^{-}\to L^{-}+{\mathrm{HO}}_{2}{}^{-}\;$$

(7) $$\;L^{-}+O_{2}{}^{-}\to {\mathrm{LO}}_{2}{}^{2-}\;$$

(8)
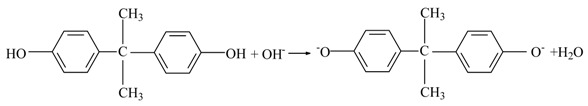

(9)
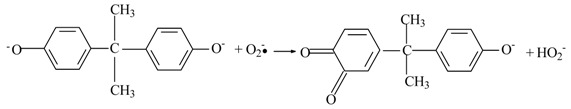

(10)
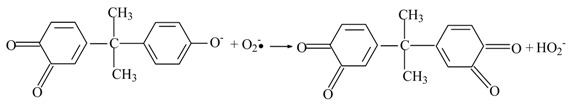

(11) $${\mathrm{LO}}_{2}{}^{2-}\to N_{2}+{\mathrm{AP}}^{2-*},$$

(12) $${\mathrm{AP}}^{2-*}\to {\mathrm{AP}}^{2-*}+\mathrm{hv}.$$

The symbols LH_2_ and AP^2−^* in the above equations refer to luminol and the excited state aminophthalate dianion, respectively. LH^−^, L^−^ and LO_2_^2−^ represent the intermediates in the reactions. The above CL and fluorescence results suggested that when BPA was injected into the system, BPA was oxidized into an intermediate quinones by O_2_^−^-induced radical oxidation. Then the O_2_^−^ are competitively consumed by BPA, less L^−^ and LO_2_^2−^ are produced, and the CL was greatly inhibited. Consequently, BPA shows a sensitive inhibition effect on the CL reaction and can be detected based on this inhibition mechanism. This result was in accord with the recorded reference [48]. 

#### 3.4.2. Analytical Calibration Curve and Limit of Detection (LOD)

**Figure 7:**
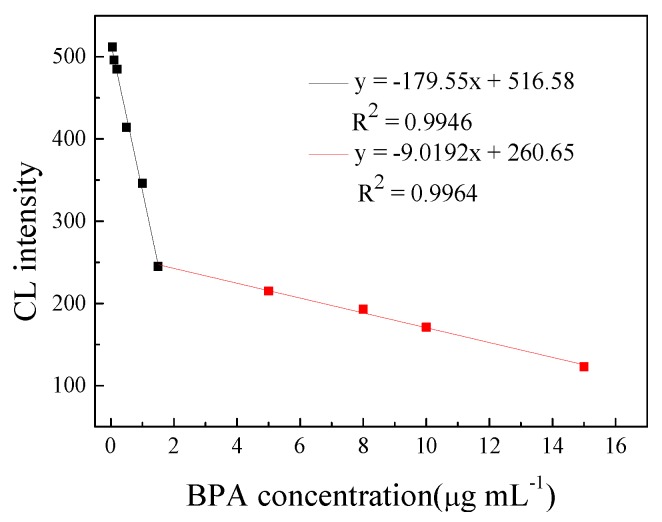
The linear response for different BPA concentration.

Under the above optimal conditions, the analytical performance was carried out with 50.0 mg MIP packed in the flow cell. As discussed in Section 3.1, there are two types of binding sites on MIMS for BPA, with lower affinity binding sites and higher affinity binding sites. As a result, the two binding sites brought two linear regions. As shown in Figure 7, the CL intensity showed two linear ranges with the BPA concentrations. The first linear range was from 50 to 15 ng mL^−1^ BPA concentrations and the detection limit was 8 ng mL^−1^ (3σ). The regression equation was *I* = −179.55*C* + 516.58 (*C* being the BPA concentration (μg mL^−1^)) with a correlation coefficient of 0.9946. The second linear range was from 15 to 150 ng mL^−1^ BPA concentrations and the detection limit was 80 ng mL^−1^ (3σ). The regression equation was *I* = −9.0192*C* + 260.65 (*C* being the BPA concentration (μg mL^−1^)) with a correlation coefficient of 0.9964. 

#### 3.4.3. Repeatability and Response Time

By testing a standard BPA solution six times, the method repeatability was measured and the relative standard deviation (R.S.D.) obtained was 2.6%.

The short response time is an analytical parameter which is commonly desirable for applications. Generally speaking, the time required for 90% change in the response of equilibrium value was defined as the response time and called *t*_90_. Experimental results indicated that *t*_90_ was 45 s under the selected conditions. Such a short response time made the proposed method preferable for the BPA determination compared with the other traditional time-needed methods. 

#### 3.4.4. Interference Study and Recovery Test

In order to evaluate the overall selectivity of the proposed method for BPA determination, influences of some ions commonly existing in water were investigated according to the recommended procedure. A 50 ng mL^−1^ BPA solution was analyzed by being added with interfering species. The tolerable limit of interfering ions (Table 1) was taken as a relative error less than 5%. As could be seen, the tolerable limit of CL detection by adopting MIMS was much higher than that without MIMS for the common cations and anions. Most of ions tested did not interfere with determination, except Fe^3+^, which could be easily eliminated using EDTA. These results showed that MIMS can be used as biomimetic recognition material in the CL analysis and improve the selectivity of the CL method. However, because no porogen was used for the preparation of MIMS, the MIMS showed indiscriminate adsorption with some structural analogues of BPA, especially for BPF. Thus, porogen should be used in the subsequent work in MIP preparation. 

#### 3.4.5. Analytical Application of the Proposed Method for BPA Determination

The proposed method was used to detect BPA in water samples. Four real water samples (including commercial drinking water, boiling water, tap water and river water) were detected by the present CL method based on the MIMS recognition. The water samples were first pretreated by filtrating with 0.45 μm Millipore filters and then a known amount of BPA was added to water samples. The recoveries were calculated from the typical standard calibration graph in triplicate and listed in Table 2. As it can be seen, the recoveries were in the range between 96–110%, indicating that the proposed method had excellent accuracy.

## 4. Conclusions

In this work, the BPA-MIMS were fabricated and used as a biomimetic recognition material for BPA determination. Due to the special binding sites on the MIMS, the BPA could be absorbed in situ on the MIMS and determined by the CL analysis with improved selectivity and sensitivity. The distinguished advantages of the proposed CL method for BPA determination based on MIMS recognition are shown below.

First, Pickering emulsion was combined with MIT for the preparation of BPA-MIMS. By locating the stabilizing SiO_2_ NPs with proper hydrophilic-hydrophobic properties at oil/water interfaces, the coalescence was successfully prevented and the droplets were stabilized. As a result, the synthesized MIMS could achieve high yields of polymer and good control of particle sizes compared with traditional bulk polymerization.

Second, the structure of the BPA templates adsorbed on the MIMS would be changed after having a CL reaction with the KIO_4_-luminol system. Then the binding sites between the BPA template and the MIMS would be destroyed and the reacted templates could be easily taken away from the MIMS. During this process, the CL reagents were not only used as detection and sensing reagents, but also as extraction eluents. Organic reagents and buffer solutions, which generally would greatly affect CL reaction, were successfully avoided from being used as the eluents to extract the BPA template from MIMS. 

Third, by packing the MIMS into the CL detection cell, the BPA could be selectively adsorbed on the MIMS and determined in situ through the CL prohibition. Then elements of BPA adsorption and extraction, together with recognition and sensing, were all integrated on the MIMS at the same time. As a result, extra steps were avoided and the analysis procedures were greatly simplified.

Finally, when MIMS were used as the biomimetic recognition element, the selectivity of the CL analysis was obviously improved due to the recognition specificity of MIMS and the sensitivity was greatly enhanced due to the enrichment effect of MIMS. As a result, an improved CL method based on MIMS recognition was successfully proposed in this work and could be applied to real sample determination with excellent selectivity and sensitivity.

## Figures and Tables

**Table 1:** Tolerable ratio of structural analogues and some interfering species to BPA with and without MIP.

Interfering substances	Without MIMS	With MIMS
SO_4_^2−^	100	200
CO_3_^2−^	10	200
Cl^−^	200	400
PO_4_^3−^	10	200
Na^+^	200	400
Ca^2+^	10	200
Mg^2+^	20	200
NH_4_^+^	10	200
Fe^3+^	10	10
citric acid	10	100
4,4′-Dihydroxybiphenyl	5	20
4,4′-Cyclohexylidenebisphenol	6	20
Bisphenol A bis(chloroformate)	5	20
Bisphenol A acetate propionate	8	15
2,2-Bis(4-hydroxy-3-methylphenyl)propane	5	10
Bis(2-hydroxyphenyl)methane	5	10
Bis(4-hydroxyphenyl)methane (BPF)	2	3
tyrosine	8	20
fulvic acid	10	50
urea	10	100

**Table 2:** Results of recovery tests on water samples.

Water sample	BPA Added (μg mL^−1^)	BPA Founded (μg mL^−1^)	Recovery (%)
River water	0	0.03	-
	0.5	0.58	110
	1	1.09	106
Tap water	0	0	-
	0.5	0.51	102
	1	1.03	103
Boiled water	0	0	-
	0.5	0.52	104
	1	0.96	96
Drinking water	0	0	-
	0.5	0.49	98
	1	0.98	98

## Supplementary Materials

The following are available online at http://www.mdpi.com/2073-4360/10/7/780/s1, Figure S1: Chemiluminescence measurements flow system. a: Luminol; b: KIO_4_; c: NaOH; d: BPA/H_2_O; Figure S2: Conditions optimization for CL measurement. (**a**) KIO_4_ concentration; (**b**) luminol concentration; (**c**) NaOH concentration; (**d**) BPA adsorption time.
