# Supplementary material for: Preparation of Molecularly Imprinted Microspheres as Biomimetic Recognition Material for In Situ Adsorption and Selective Chemiluminescence Determination of Bisphenol A
Source: Polymers (Basel). 2018 Jul 16;10(7):780. doi: 10.3390/polym10070780 (PMC6403925; doi:10.3390/polym10070780)
Supplement: Supplementary file 1 [file polymers-10-00780-s001.pdf]

Supporting Information

Preparation of Molecularly Imprinted Microspheres as Biomimetic Recognition Material for In Situ Adsorption and Selective Chemiluminescence Determination of Bisphenol A

Yan Xiong [1,2,*], Qing Wang [1], Ming Duan [1,2,*], Jing Xu [3], Jie Chen [1] and Shenwen Fang [1,2]

[1] School of Chemistry and Chemical Engineering, Southwest Petroleum University, Chengdu 610500, China; qwang@163.com (Q.W.); jiechen92@163.com (J.C.); swhen@163.com (S.F.)

[2] Oil and Gas Field Applied Chemistry Key Laboratory of Sichuan Province, Southwest Petroleum University, Chengdu 610500, China

[3] Liaoning Entry-Exit Inspection and Quarantine Bureau, Dalian 116001, China; jingxu99@163.com

* Correspondence: xiongyan207@163.com (Y.X.); mduan124@swpu.edu.cn (M.D.); Tel.: +86-28-83037346 (Y.X.)

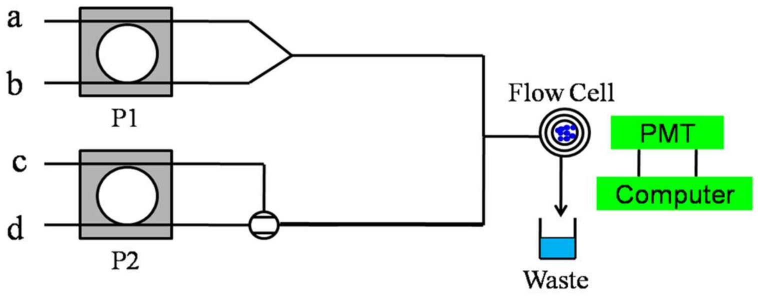

Figure S1. Chemiluminescence measurements flow system. a: Luminol ; b:KIO_4; c:NaOH; d:BPA/H_2O.

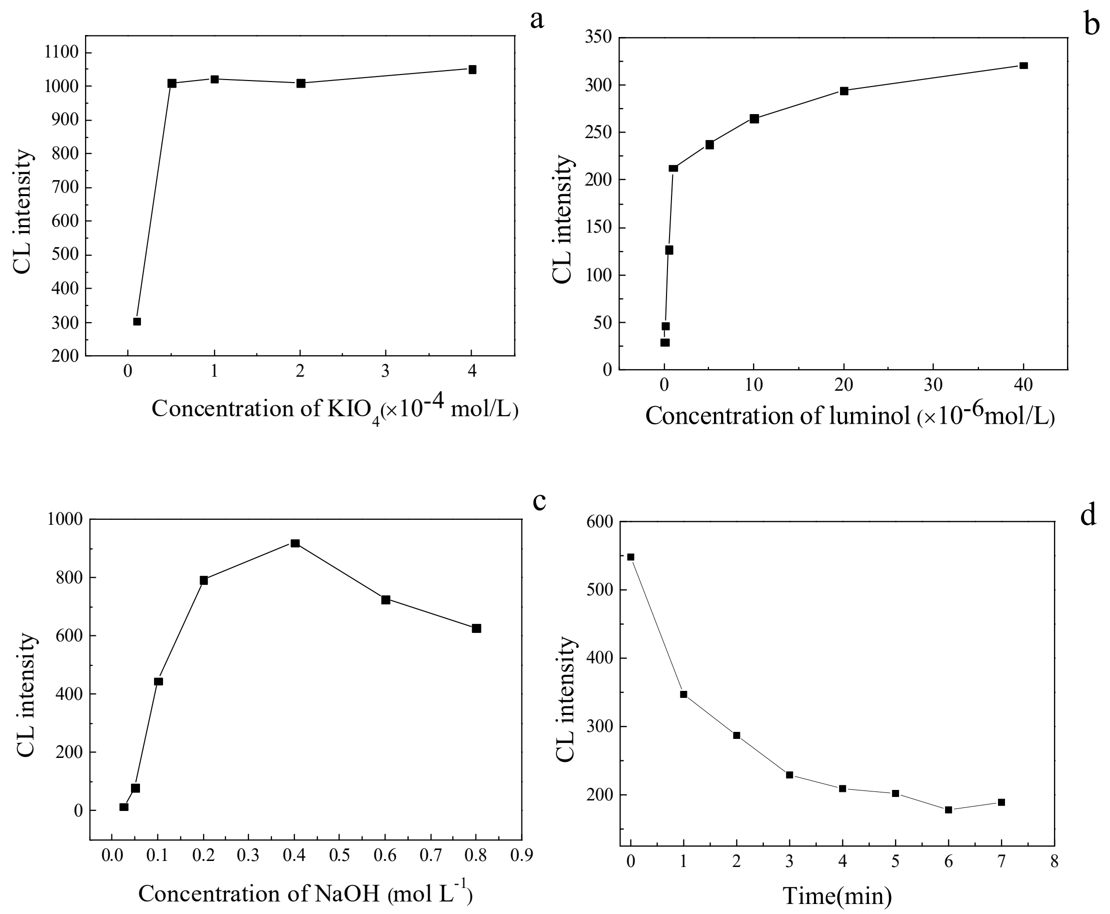

Figure S2. Conditions optimization for CL measurement. (**a**) KIO_4 concentration; (**b**) luminol concentration; (**c**) NaOH concentration; (**d**) BPA adsorption time.
